# Crossbreeding Simmental with Mongolian, and Holstein cattle can improve feed efficiency and energy metabolism by upregulating COX3 and downregulating PRSS2 gene expression

**DOI:** 10.3389/fnut.2025.1524242

**Published:** 2025-02-21

**Authors:** Yi Wu, Pengfei Zhao, Xiaorui Li, Mingke Huangfu, Zhimeng Chen, Chunjie Wang, Hao Chen, Aorigele Chen

**Affiliations:** ^1^College of Animal Science, Inner Mongolia Agricultural University, Hohhot, China; ^2^College of Veterinary Medicine, Inner Mongolia Agricultural University, Hohhot, China

**Keywords:** energy metabolism, cattle, breed, RNA sequencing, glucose

## Abstract

The selective breeding of beef cattle plays an important role in meeting the growing demand for beef and improving production performance. This study used fattened cattle of the Simmental (S) breed, and two crossbreeds: Simmental × Mongolian (SM) and Simmental × Holstein (SH), which were healthy, of similar age and weight. The results showed that the blood glucose (GLU) levels of the crossbred, genetically improved SM and SH groups were higher than that of the S group. Compared with the S group, there were 49 differentially expressed genes (DEGs) in the SM group, of which 18 genes were up-regulated and 31 genes were down-regulated; and 1,031 DEGs in the SH group, of which 251 genes were up-regulated and 780 genes were down-regulated. We found that crossbreeding may increase GLU levels in the blood by upregulating cytochrome C oxidase subunit 3 (COX3) gene expression and downregulating of PRSS2, providing glycogen to the organism, and therefore enhancing GLU-converting capacity. This study highlighted the differences in feed utilization and energy metabolism among crossbred breeds and provides theoretical support for crossbreeding as a means of selecting breeds and improving beef cattle production. However, the expression of the genes were not validated in the present experiments, and these results need further validation.

## 1 Introduction

It is well known that beef accounts for a large proportion of the red meat consumed by humans globally (1). In recent decades, researchers have focused on selecting early maturing cattle breeds and performing interbred crosses to improve carcass growth performance (2). Excellent foreign breeds of cattle have been introduced to China since the 1960s, and crossbreeding of these breeds with native breeds has significantly improved beef production; currently crossbreeds are reared in Inner Mongolia, Xinjiang, Heilongjiang, Shanxi, Hebei, and other areas (3). Therefore, it is important to develop breeding programs to meet the growing demand for beef and to improve energy efficiency (4).

Energy metabolism is a complex process of converting chemical energy from nutrients such as sugars, lipids, and proteins into heat and adenosine triphosphate (ATP) (5). The main sources of energy for the organism of the animal are glycogen and lipids. Glycogen is a large polymer made up of multiple glucose units. Glucose in the blood is taken up by histiocytes through glycolysis (6). It has been shown that enhanced glycolysis and pyruvate metabolism *in vivo* increase energy metabolism activity (7). Lipids are divided into fats, phospholipids and sterols. Cholesterol (TC) is an important raw material in the cell and is involved in the synthesis of cell membranes, bile acids and vitamin D. Apolipoproteins are responsible for transporting cholesterol in the serum (8). Very low density lipoprotein (VLDL) is synthesized in the liver (9). During metabolism, VLDL completes its exchange with cholesteryl esters and is converted to LDL, which awaits long-term energy supply and is partially retransported to the liver (10). Low-density lipoproteins (LDL) are responsible for transporting cholesterol to peripheral tissues throughout the body and are themselves rich in cholesterol (11). Excess free cholesterol in cells of peripheral tissues can be actively or passively transported to lipid-free or small amounts of lipid-containing apolipoprotein A-I (ApoA-I), which is synthesized by the liver to form the initial HDL particles, and circulating systemic HDL-C can be selectively taken up by the intrahepatic HDL receptor (scavenger receptor class20BtypeI, SR-BI) and HDL particles are ultimately transported from peripheral tissues back to the liver and excreted into the bile, a process also known as reverse cholesterol transport (12).

Studies have shown that the expression patterns of genes in different tissues help elucidate the evolutionary mechanisms and biological functions of organisms (13). In particular, the differences in gene expression patterns in different tissues can provide valuable clues for understanding breed formation and genetic pathways in cattle. DEGs or core driver genes have been identified as potential candidates involved in important functions, such as growth and development (14), meat quality (15), hair follicles (16) and disease resistance (17). Huang et al. (18) confirmed a positive correlation between intramuscular fat content and the expression of phosphoenolpyruvate carboxykinase 1 (PCK1) in buffaloes through RNA sequencing analysis. The researchers confirmed that seven genes (HSPA12A, HSPA13, PPARγ, MYL2, MYPN, TPI and ATP2A1) influence water holding capacity, providing important insights into the molecular mechanisms of water holding capacity (19). Specific high expression of genes by RNA sequencing of subcutaneous adipose tissue of Qinchuan and Angus × Qinchuan cattle demonstrates that crossbreeding improves beef production (20). However, most of these previous studies on cattle have been carried out in dairy cows, with limited studies on beef cattle (21). Therefore, there is an urgent need for comparative analyses of tissue-specific expression patterns based on the beef cattle transcriptome to functionally annotate the important genes in beef cattle.

Therefore, the aim of this study was to perform a differential gene expression analysis of the transcriptomic data generated from the dorsal longissimus dorsi muscle (a metabolically active muscle that is economically important (22)) of beef cattle from different crossbreeds fed the same dietary formulations. Additionally, gene enrichment and correlation analyses were performed to reveal differences in energy metabolism of the crossbreeds. This study provides theoretical support for crossbreeding in order to improve genetics and increase beef cattle production.

## 2 Materials and methods

This study was conducted in Xing’an League, Inner Mongolia Autonomous Region, China. All of the study procedures were reviewed and approved by the Institutional Animal Care and Use Committee of Inner Mongolia Agricultural University, Hohhot, China (protocol number 2020079) and were in accordance with the animal welfare guidelines of the China Animal Welfare Committee. The use of animals and private land in this study was approved by their legal owners. Transportation and sampling were performed in accordance with Chinese laws and local regulations, and we confirmed that the authors complied with the Animal Research Reporting of *In Vivo* Experiments (ARRIVE) guidelines.

### 2.1 Animals and feeding management

In this study, we have used a total of 18 cattle belonging to Simmental (S), Simmental x Mongolian (SM) and Simmental x Holstein (SH), taking 6 animals from each group. All healthy and of similar age (480.33 ± 43.27 days) and weight (573.00 ± 30.60 kg). All groups were fattened at the Tianmuzhen Farm in Xing’an League, Inner Mongolia. The experimental cattle were housed in mixed pens and fed a total mixed ration twice daily at 08:00 and 18:00 h, with free access to feed and water during the 180 d fattening period. They were fed using the same feeding strategy (see Table 1) during fattening, the three head in each group closest to the average weight were selected for slaughter and the cattle were slaughtered at the slaughterhouse of Inner Mongolia Tianmuzhen Meat Industry Co.

**Table 1:** Composition and nutrient levels of experimental diets.

Items	%
Ingredients,% of DM	
Dry rice straw	31.50
Corn silage	18.50
Corn	20.80
Wheat bran	14.80
Soybean meal	12.60
CaHPO4	0.30
NaCl	0.50
Premix^1^	1.00
Total	100.00

^1^Premix contains (per kg premix) 100,000 IU vitamin D3, 4,000 IU vitamin E, 380,000 IU vitamin A, 2560 mg Fe, 2,560 mg of Fe, 790 mg of Cu, 3,000 mg of Zn, 140 g of Ca, 28 g of P, 1,680 mg of Mn, 30 mg of Se, 28 mg of I and 18 mg of Co.

### 2.2 Determination of the blood indices

Before slaughter, blood was collected from each cattle after fasting in non-anticoagulated and anticoagulated blood collection tubes from nine cattle and allowed to stand for 30 min, Centrifugation in a centrifuge (Centrifuge 80-2, Jiangsu Kangjie Medical Devices Co., Ltd. China) at 1076.7 rcf for 10 min to separate the serum and plasma, which were then divided into centrifuge tubes and stored at −80°C for the determination of serum biochemical indices (three repetitions per cattle). The levels of glucose (GLU), triglycerides (TG), total cholesterol (TC), high-density lipoproteins (HDL), very low-density lipoproteins (VLDL) and beta-hydroxybutyric acid (BHBA) were determined using Bovine ELISA kits (HDL:CK-E150097B;BHBA:CK-E92679B;VLDL:CK-E150082-48;GLU:CK-E95613B-48;TG:CK-E90126B;TC:CK-E93389B) purchased from Quanzhou Ruixin Biotechnology Co., Ltd. (China) according to the manufacturer’s instructions.

### 2.3 Determination of fatty acid content in dorsal longissimus dorsi muscle

Nine 50 mg samples from each group were placed in a 2 mL grinding tube with steel beads and 1 mL of dichloromethane: methanol (1:1),and the sample was then ground in a freezer grinder. The sample was then sonicated for 15 min at 4°C, followed by 15 min of standing at −20°C. The supernatant was removed by centrifugation (Eppendorf, Centrifuge 5,424 R,MJPRO-YQ-015) at 13,000 rcf for 10 min and dried under nitrogen atmosphere. Then, 0.5 mL of sodium hydroxide in methanol (0.5 mol/L) was added, vortexed for 30 s, and sonicated at 60°C for 30 min in a water bath. After cooling, 0.5 mL of hexane was added, vortexed for 30 s, centrifuged at 4°C for 10 min, and 100 μL of the upper layer (hexane layer) was transferred to the injection vial and detected by GC–MS (23) using an 8890B-5977B (Agilent Technologies Inc. CA, United States) gas chromatograph. The default parameters of the Masshunter quantification software (Agilent, United States, v10.0.707.0) were used for the automatic identification and integration of each ionic fragment of the target fatty acids with the aid of manual inspection. A linear regression standard curve was plotted, with the analyte concentration as the horizontal coordinate and the mass spectral peak area as the vertical coordinate. The concentration of each sample was calculated by substituting the value into a linear equation.

### 2.4 Total RNA extraction, library construction, and RNA sequencing

Total RNA was extracted from the dorsal longissimus dorsi muscle tissues using TRIzol reagent (catalog number 15596026, Invitrogen, Carlsbad, CA, United States), according to the method described by Hao et al. (24). RNA quality was determined using a 5,300 Bioanalyzer (Agilent, Santa Clara, CA, USA) and quantified using an ND-2000 (NanoDrop Technologies). Only high-quality RNA sample (OD260/280 = 1.8 ~ 2.2, OD260/230 ≥ 2.0, RIN ≥ 6.5, 28S:18S ≥ 1.0, >1 μg) was used to construct sequencing library. Using the eukaryotic A-tail structure, A-T base pairing was performed using oligomeric (dT) magnetic beads complementary to the A-tail (Illumina® Stranded mRNA Prep, Ligation, Illumina, United States). mRNA was isolated for transcriptome analysis, and DNA was digested with DNase I (TaKara) (Tiangen, Shanghai, China). Randomly interrupted mRNA, a small fragment of approximately 300 base pairs (bp) was isolated using magnetic bead screening (Illumina® Stranded mRNA Prep, Ligation, Illumina, United States). The mRNA was used as a template, and the cDNA strand was synthesized using a 6-base random primer. Double-stranded cDNA was purified using a double-stranded cDNA was synthesized using a SuperScript double-stranded cDNA synthesis kit (Invitrogen, CA) with random hexamer primers (Illumina, United States); the purified double-stranded cDNA was repaired, attached to the a-tail and ligated to the sequencing connector. Library construction and RNA sequencing were performed by Shanghai Majorbio Bio-Pharm Biotechnology Co., Ltd. (Shanghai, China). cDNA target regions of 300 bp were selected from the libraries using 2% low-range ultra agarose, followed by PCR amplification using Phusion DNA polymerase (NEB) for 15 PCR cycles. The paired-end RNA-seq library was sequenced using a NovaSeq 6,000 sequencer (2 × 150 bp read length) after quantification using Qubit 4.0.

### 2.5 Sequencing data analysis

The raw sequencing data were subjected to quality control and the reads were mapped using fastp (25), The specific steps and sequences are as follows: (1) Remove the splice sequences from the reads, and remove the reads that do not have fragments inserted due to splice self-linkage and other reasons; (2) Trim off the low-quality (quality value less than 20: calculate the average quality value according to the sliding window size of 4) bases at the first end of the sequence (5″ end), and trim off the low-quality (quality value less than 3) bases at the end of the sequence (3″ end); (3) Removal of reads containing N (fuzzy bases): reads with more than 5 N are discarded; (4) Discard sequences less than 30 bp in length after de-adapter and quality trimming. After the completion of data quality control, the data after quality control were again subjected to statistics and quality assessment, which also included: base error rate distribution statistics and base content distribution statistics (Table 2).

**Table 2:** QC data for each sample.

Sample^1^	Raw reads	Raw bases	Clean reads	Clean bases	Error rate (%)	Q20 (%)	Q30 (%)	GC content (%)
MR3	59099368.00	8924004568.00	58609182.00	8677007196.00	0.03	97.98	93.76	55.99
MR2	59513904.00	8986599504.00	59102844.00	8737870865.00	0.03	98.03	93.88	55.18
MR1	60235454.00	9095553554.00	59784836.00	8823030875.00	0.03	98.08	94.01	54.13
HR3	60667538.00	9160798238.00	59090288.00	8750131480.00	0.03	97.76	93.28	55.30
HR2	60078966.00	9071923866.00	58943744.00	8634845344.00	0.03	97.99	93.89	56.36
HR1	54481214.00	8226663314.00	53435206.00	7816957530.00	0.03	97.99	93.81	53.73
SR3	63027642.00	9517173942.00	61661372.00	8982864673.00	0.03	97.87	93.51	53.77
SR2	59534818.00	8989757518.00	58972742.00	8614789172.00	0.03	97.93	93.66	53.68
SR1	63733102.00	9623698402.00	62430574.00	9027073876.00	0.03	97.99	93.88	56.22

^1^Sample: the name of the sample; Raw reads: the total number of entries of the original sequencing data (reads, on behalf of the sequencing reads, a reads that is one); Raw bases: the total amount of original sequencing data (i.e., the number of Raw reads multiplied by the length of reads); Clean reads: the total number of entries of the post-quality-control sequencing data; Clean bases: the total amount of post-quality control sequencing data (i.e., the number of clean reads multiplied by the length of reads); Error rate (%): the average error rate of sequencing bases corresponding to the quality control data, which is generally below 0.1%; Q20 (%), Q30 (%): the quality evaluation of post-quality control sequencing data, with Q20 and Q30 referring to the quality of the sequencing data, respectively. Q20 (%) and Q30 (%): quality assessment of the sequencing data after quality control, Q20 and Q30 refer to the percentage of total bases with sequencing quality of 99 and 99.9% or more, generally Q20 is above 85% and Q30 is above 80%; (8) GC content (%): the percentage of total bases corresponding to the G and C bases of the quality control data.

As described by Man et al. (26). Subsequently, the mapped reads were individually aligned to the *Bos taurus*^1^ genome using HISAT2 software (27). Finally, the reads were then assembled using StringTie using the method described in Pertea et al. (28) RSEM (29) software was used to quantify the gene and transcript levels. DESeq2 (30) was used to calculate differential expression with a threshold of (|log2FC| ≥ 1 and FDR < 0.05). GO and KEGG (31–33) were used for functional enrichment analyses to identify significantly enriched DEGs in metabolic pathways and GO terms.

### 2.6 Data statistics and analyses

Statistical analyses were performed using SPSS (34) software (SPSS v. 21, SPSS Inc.; Chicago, IL, United States). Significance of serum biochemical parameters and fatty acid contents. Statistical significance was determined by one-way analysis of variance (ANOVA), followed by the LSD multiple comparison test. The results in this study were presented as the means ± standard deviation (SD). *p* < 0.05 was considered statistically significant and indicated by “*,” whereas “**” indicated *p* < 0.01.

## 3 Results

### 3.1 Comparison of the serum biochemical parameters

**Figure 1:**
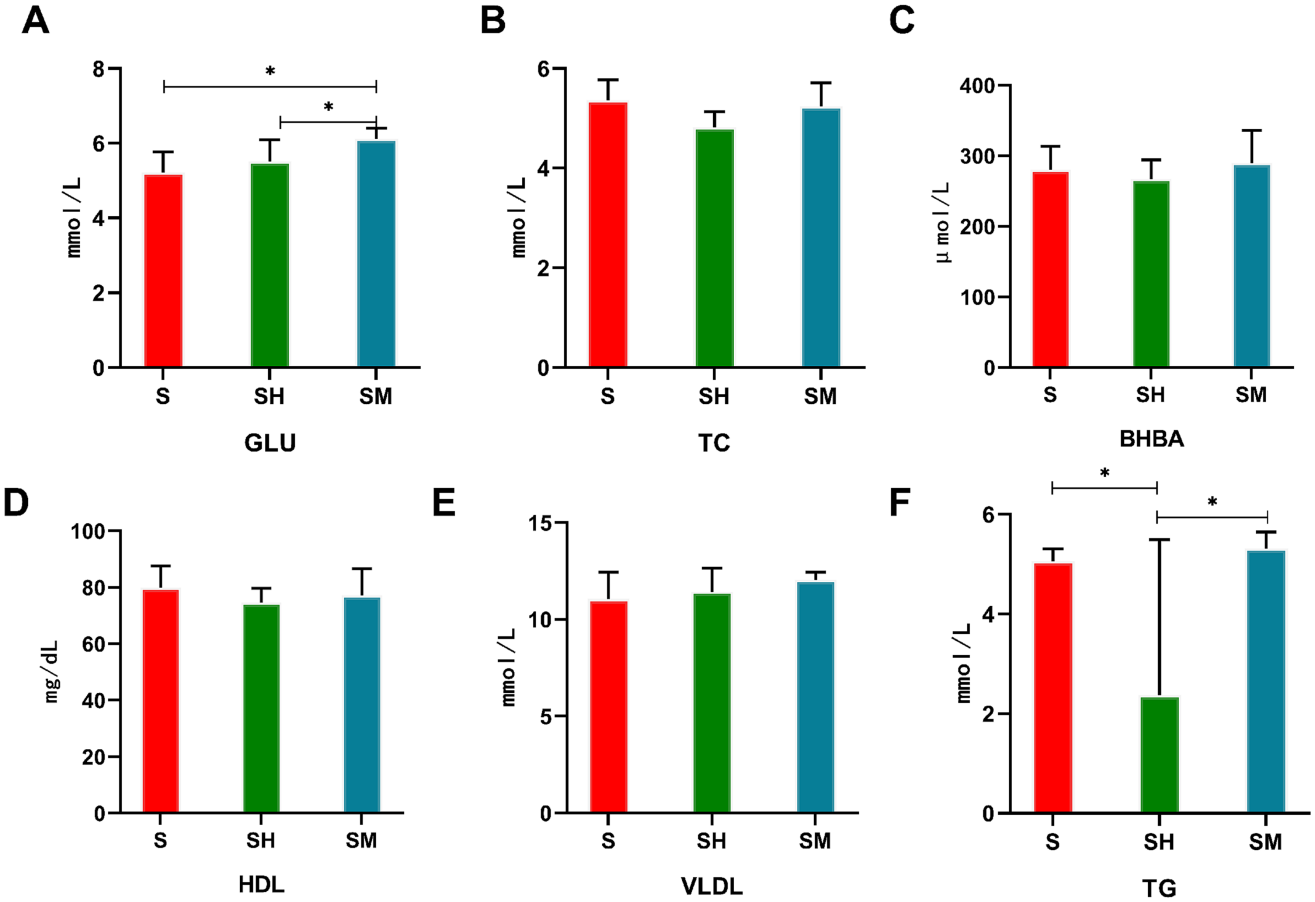
Comparison of the serum biochemical indices of Simmental (S), Simmental × Mongolian (SM) and Simmental × Holstein (SH) cattle.”*” represents *p* < 0.05. GLU: triglycerides **(A)**, TC: total cholesterol **(B)**, BHBA: beta-hydroxybutyric acid **(C)**, HDL: high-density lipoprotein **(D)**, VLDL: very low-density lipoprotein **(E)**, TG: Glucose **(F)**.

When comparing the blood serum indices between the different groups, as shown in Figure 1, the serum TG content in both the SM and S groups was significantly higher than that in the SH group (*p* < 0.05), the GLU content in the SM group was significantly higher than that in the S group (*p* < 0.05), and the differences in serum TC, BHBA, HDL, and VLDL contents between groups were not significant (*p* > 0.05).

### 3.2 Determination of the fatty acid content in dorsal longissimus dorsi muscle

The fatty acid contents of the dorsal longissimus dorsi muscles of the three groups of fattened cattle are shown in Table 3. There were 34 fatty acids detected in all three groups in total, which were classified by saturation. The contents of SFAs, unsaturated fatty acids (UFAs), monounsaturated fatty acids (MUFAs), and polyunsaturated fatty acids (PUFAs) were determined separately using GC–MS. The results showed that pentadecanoic acid and linoleic acid contents in the dorsal longissimus dorsi muscle of the SM group were found to be significantly higher than those of the S and SH groups (*p* < 0.05). The Docosanoic acid content in the S group was significantly higher than that in the two crossbreed groups (*p* < 0.01). The erucic acid contents in the dorsal longissimus dorsi muscles of the S and SM groups were found to be significantly higher than those in the SH group (*p* < 0.01). Eicosatrienoic acid levels in the S and SM groups were significantly higher than those in the SH group (*p* < 0.05). The UFA and MUFA contents in the SM group were significantly higher than those in the S and SH groups (*p* < 0.05).

**Table 3:** Fatty acid contents of the different groups.

Fatty acid (μg/mg)	S^1^	SH^1^	SM^1^	*p*- value
C12:0	0.01 ± 0.00^b^	0.01 ± 0.00^b^	0.02 ± 0.01^a^	0.03
C14:1	0.04 ± 0.00	0.03 ± 0.00	0.05 ± 0.01	0.10
C15:1	0.65 ± 0.29^b^	0.53 ± 0.33^b^	4.46 ± 2.14^a^	0.01
C18:1n6t	0.04 ± 0.00	0.04 ± 0.00	0.05 ± 0.01	0.33
C18:1n9t	0.05 ± 0.00	0.05 ± 0.00	0.07 ± 0.02	0.27
C18:1n11c	0.13 ± 0.03	0.10 ± 0.03	0.21 ± 0.11	0.22
C19:1n10t	0.04 ± 0.00	0.04 ± 0.00	0.04 ± 0.00	0.11
C18:2n6c	1.10 ± 0.12	0.74 ± 0.11	1.15 ± 0.34	0.11
C20:0	0.04 ± 0.00	0.03 ± 0.00	0.04 ± 0.00	0.09
C20:1 T	0.02 ± 0.00	0.01 ± 0.00	0.02 ± 0.00	0.09
C18:3n6	0.0512 ± 0.0021^b^	0.0476 ± 0.0011^b^	0.0476 ± 0.0005^a^	0.029
C18:3n3	0.06 ± 0.00	0.06 ± 0.00	0.06 ± 0.00	0.34
C22:0	0.02 ± 0.00^a^	0.02 ± 0.00^b^	0.02 ± 0.00^b^	0.01
C22:1	0.05 ± 0.00 ^a^	0.04 ± 0.00^b^	0.06 ± 0.00^a^	0.01
C20:3n6	0.06 ± 0.00 ^a^	0.05 ± 0.00^b^	0.06 ± 0.00^a^	0.01
SFAs	2.18 ± 0.28	1.50 ± 0.53	3.69 ± 2.37	0.23
UFAs	4.51 ± 0.418^b^	2.74 ± 0.40^b^	9.91 ± 2.79^a^	0.00
MUFAs	2.91 ± 0.34^b^	1.59 ± 0.44^b^	8.28 ± 2.53^a^	0.00
PUFAs	1.60 ± 0.13	1.15 ± 0.12	1.63 ± 0.37	0.08

^1^ S: Simmental, SM: Simmental × Mongolian and SH: Simmental × Holstein. Superscript a or b in the same row indicate significant difference (*p* < 0.05).

### 3.3 Quality control of transcriptome RNA-sequencing data

As shown in Table 4, after matching the clean reads of each sample to the reference genome, 4.39–5.19% and 91.45–92.48% of the clean reads for each sample were repeatedly and uniquely mapped, respectively, with an average match rate of 96.68%.

**Table 4:** Sequencing comparison results.

Sample^1^	Total reads	Total mapped	Multiple mapped	Uniquely mapped
S1^2^	61,661,372	59,579,274 (96.62%)	3,061,496 (4.97%)	56,517,778 (91.66%)
S2^2^	58,972,742	56,878,669 (96.45%)	2,782,596 (4.72%)	54,096,073 (91.73%)
S3^2^	62,430,574	60,379,294 (96.71%)	3,155,623 (5.05%)	57,223,671 (91.66%)
SH1^2^	59,090,288	56,924,931 (96.34%)	2,592,110 (4.39%)	54,332,821 (91.95%)
SH2^2^	58,943,744	57,107,391 (96.88%)	2,849,691 (4.83%)	54,257,700 (92.05%)
SH3^2^	53,435,206	51,891,001 (97.11%)	2,473,300 (4.63%)	49,417,701 (92.48%)
SM1^2^	58,609,182	56,353,946 (96.15%)	2,753,224 (4.7%)	53,600,722 (91.45%)
SM2^2^	59,102,844	57,263,439 (96.89%)	2,740,933 (4.64%)	54,522,506 (92.25%)
SM3^2^	59,784,836	57,977,774 (96.98%)	3,105,278 (5.19%)	54,872,496 (91.78%)

^1S: Simmental group; SM: Simmental × Mongolian group; SH: Simmental × Holstein group. 2 Numbers 1, 2 and 3 are sample numbers of the groups.^

### 3.4 DEG analysis of differentially expressed genes

**Figure 2:**
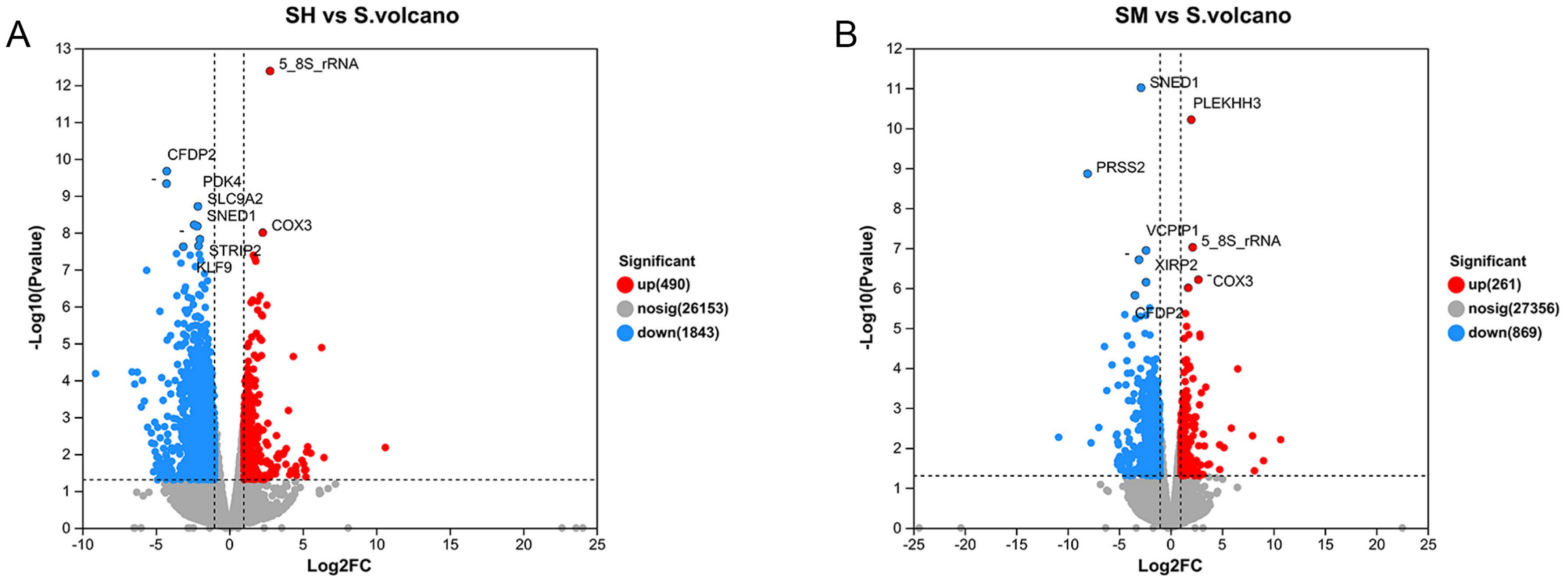
Volcano plots of SH vs. S **(A)** and SM vs. S **(B)** expression differences. Genes with *p*-values <0.01(FDR<0.05) and Fold-Change ≥2 were identified as differentially expressed genes (DEGs). Red points indicate the significantly upregulated genes, and blue points indicate the significantly downregulated genes.

Each sample was analyzed individually in order to identify DEGs. As per an empirical study by Du et al. (23), genes with *p*-values less than 0.01 and a fold change ≥2 were identified as DEGs. In the present study, 1,031 DEGs were identified between the SH and S groups. Of these, 251 genes were upregulated and 780 were downregulated, as shown in the volcano plot in Figure 2A(FC > 2, and FDR < 0.05), Specific FDR values are shown in Table 4. A total of 49 DEGs were identified between the SM and S groups, of which 18 were upregulated and 31 were downregulated, as shown in Figure 2B. Each point in the graph represents a specific gene, with the red points indicating significantly upregulated genes, blue points indicating significantly downregulated genes, and grey points indicating non-significantly different genes. After mapping all of the genes, it can be seen that the points on the left are genes that are differentially downregulated, and the points on the right are genes that are differentially upregulated; the closer to the two sides and the top the points are, the more significant the difference in expression. The genes upregulated in the SH group were 5-8-S rRNA and COX3, and the genes downregulated were CFDP2, PDK4, SLC9A2, SNED1, STRIP2 and KLF9. The genes upregulated in the SH group in the SM group were PLEKHH3, 5-8-S rRNA and COX3 and the genes downregulated were CFDP2, SNED1, PRSS2, VCPIP1,CFDP2.

### 3.5 KEGG annotation analysis of the differentially expressed genes

**Figure 3:**
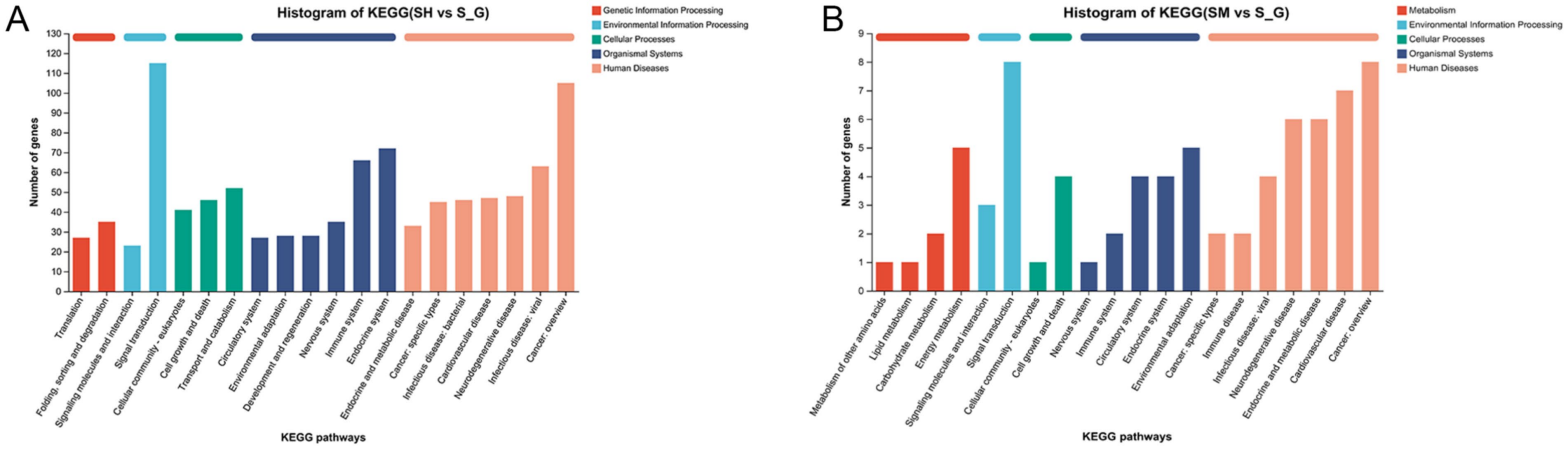
KEGG annotation map of differentially expressed genes in SH vs. S **(A)** and SM vs. S **(B)**.

The KEGG database was used to analyze gene functions, and link genomic information and functional information. Genes in the gene sets were classified according to the pathways in which they were involved or their functions. In Figure 3, the horizontal coordinate is the number of genes or transcripts in each pathway, and the vertical coordinate is the name of the pathway in the KEGG database. As shown in Figure 3, DEGs in the SH and S groups were enriched in six KEGG pathways: metabolism, genetic information processing, cellular processes, organismal systems, human diseases, and environmental information processing. DEGs in the SM and S groups were enriched in five major KEGG metabolic pathways: cellular processes, organismal systems, metabolism, human diseases, and environmental information processing.

### 3.6 KEGG enrichment analysis of the differentially expressed genes

**Figure 4:**
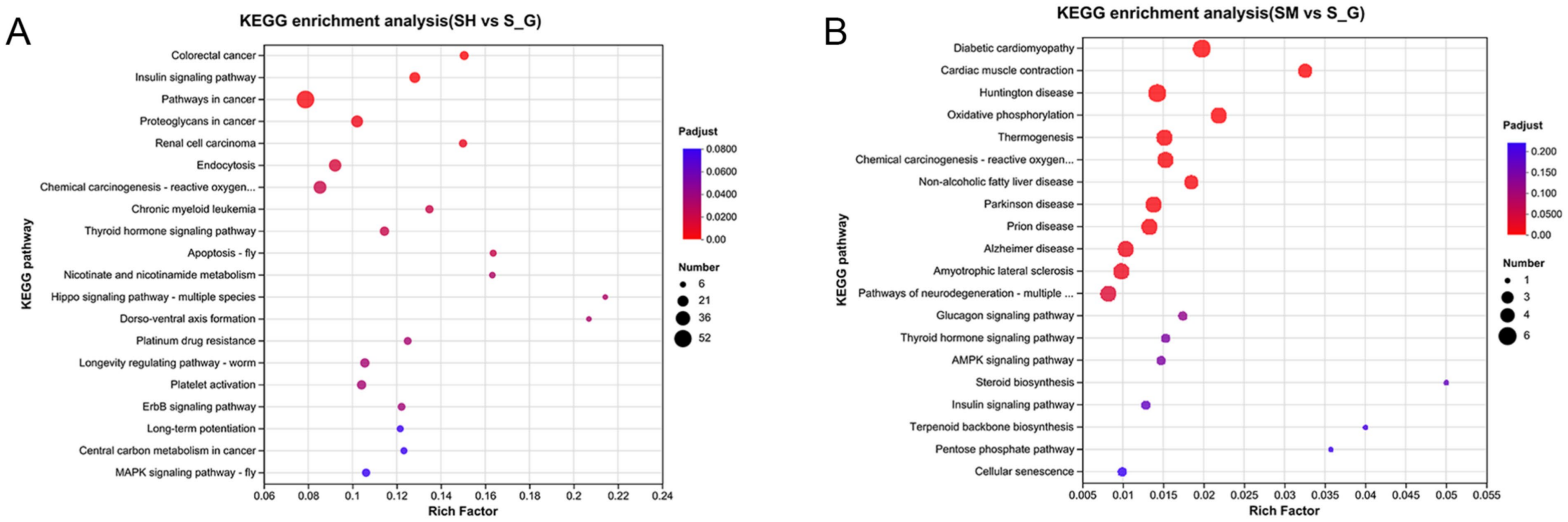
KEGG enrichment maps of DEGs in SH vs. S **(A)** and SM vs. S **(B)**. The vertical coordinate is the name of the pathway and the horizontal coordinate is the ratio of the number of genes enriched in the pathway to the number of annotated genes. The size of each dot is proportional to the number of genes in the pathway, and the color of the dots corresponds to different P-adjusted regions.

KEGG pathway enrichment analysis was performed on the DEG gene sets and two sets of gene enrichment bubbles were obtained. The vertical coordinate is the name of the pathway and the horizontal coordinate is the ratio of the number of genes enriched in the pathway to the number of annotated genes. The size of each dot is proportional to the number of genes in the pathway, and the colors of the dots correspond to different P-adjusted regions. As shown in Figure 4A, the top 20 enriched pathways in the SH and S groups were colorectal and insulin signaling pathways. The pathways that were found to be more highly enriched in the SM and S groups were oxidative phosphorylation, thermogenesis, cardiac muscle contraction, and non-alcoholic fatty liver disease (Figure 4B).

### 3.7 Correlation analysis of phenotypic indicators and differential gene datasets

**Figure 5:**
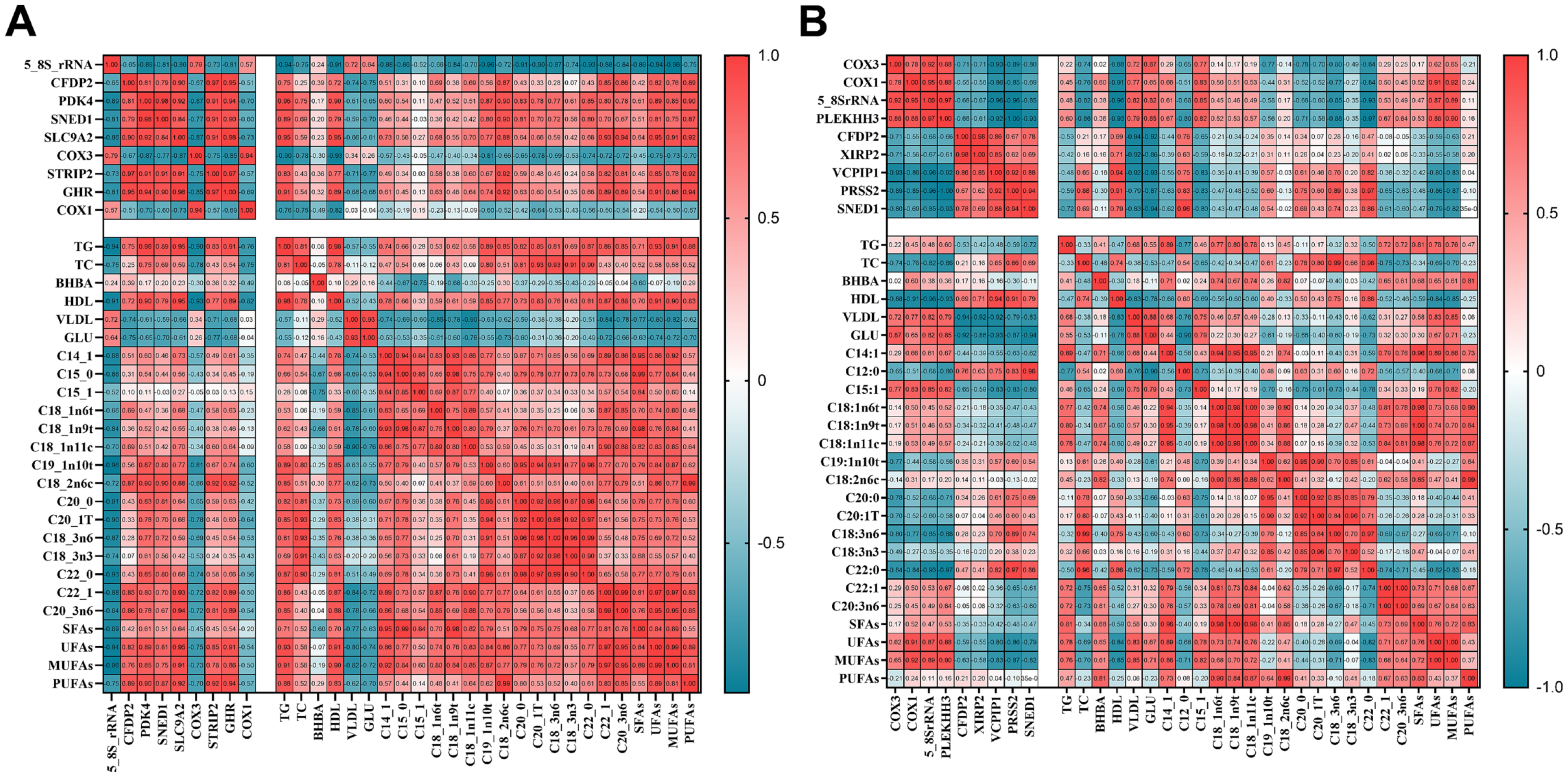
Correlation analysis of the phenotypic indicators and differential gene datasets. **(A)** Heatmap of correlation between the SH and S groups; **(B)** Heatmap of correlation between the SM and S groups.

We selected nine genes with significant differences between the SH, SM, and S groups and performed a relevance analysis of the blood biochemical parameters and fatty acid content of the longest dorsal muscle, as shown in Figure 5. In group SH, we found that HDL content was negatively correlated with the expression of 5-8-S rRNA, COX1 and COX3 and positively correlated with the expression of GHR and SLC9A2; UFA content was negatively correlated with the expression of 5-8-S rRNA and positively correlated with the expression of SLC9A2, GHR and SEND1; MUFA content was negatively correlated with the expression of 5-8-S rRNA and positively correlated with the expression of SLC9A2, GHR and SEND1.In group SM, HDL content in SM and S groups was negatively correlated with the expression of 5-8-S rRNA, COX1 and COX3; GLU levels were positively correlated with the expression of 5-8-S rRNA and COX3; UFA levels were positively correlated with the expression of 5-8-S rRNA and COX1; and MUFA levels were positively correlated with the expression of 5-8-S rRNA and COX1, and negatively correlated with the expression of SNED1.

## 4 Discussion

Blood biochemical indicators reflect changes in the metabolism, growth, and developmental status of animals (35). In our study, a total of 12 DEGs were associated with energy metabolism in group H, and 5 DEGs were associated with energy metabolism in group M. GLU is a key nutrient required for energy production in all mammals (36). Inadequate dietary energy intake can lead to decreased serum GLU concentrations and reduced nutrient utilization (37). Sugars are synthesized in cattle as energy-storing glycogens or fats through gluconeogenesis (38). In the present study, the SM group showed the highest levels of GLU, which were positively correlated with COX3 and 5–8 S rRNA (Figure 5). Cytochrome c oxidase (COX or complex IV) plays an important role in the mitochondrial respiratory chain by converting molecular oxygen into water molecules, and thus generating and storing energy through the potential difference between the inside and outside of the mitochondrial membrane (39). Therefore, crossbreeding with Mongolian cattle may have a better GLU conversion capacity by upregulating the expression of the COX3 gene, resulting in a high level of GLU in the blood, providing glycogen and fat to the body, and generating more energy available for the body; however, the mechanism by which Mongolian cattle have a higher GLU conversion capacity needs to be further investigated. Serine protease 2 (PRSS2) is a serine protease, an important member of the trypsin family and a major protein hydrolysing enzyme with serine as its active center (40). Abo-Ismail et al. (10) found that novel single nucleotide polymorphisms (SNPs) in the PRSS2 gene were associated with feed conversion efficiency in beef cattle. Abo-Ismail et al. (41) found that novel single nucleotide polymorphisms (SNPs) in the PRSS2 gene were associated with feed conversion efficiency in beef cattle. In our study, PRSS2 was negatively correlated with GLU content, and downregulation of PRSS2 increased GLU, thereby improving feed conversion efficiency. Therefore, we inferred that the downregulation of PRSS2 may have a synergistic effect with the increase in COX3, which together acted on GLU.

Fatty acids are the main components of lipids, and they can be classified as SFAs or UFAs based on the presence of double bonds in their structures. UFAs can then be classified as MUFAs or PUFAs according to the number of double bonds (42). Angus cattle were found to have a higher proportion of overall MUFAs than Charolais cattle, suggesting that different breeds have different abilities to accumulate MUFA in adipose tissues (43). Research has suggested that differences in fatty acid composition between breeds are related to the overall fat content of the carcass (44). However, De et al. (45) suggested that there might be genetic variations in fatty acid metabolism within breeds that could alter fatty acid composition. Our study found that the UFA and MUFA contents were significantly higher in the SM group than in the S and SH groups, suggesting that beef from fattened cattle after crossbreeding with Mongolian cattle has more flavor-related fatty acids and, therefore, a richer meat flavor. This is similar to the results of the study by Albuquerque et al. (46), in which changes in fatty acid composition can improved meat quality. In our experiments, muscle UFA and MUFA levels were found to be significantly higher in the SM group than in the other two groups, and both UFA and MUFA levels were significantly and positively correlated with COX1 and 5-8-S rRNA in correlation analyses. Therefore, it is reasonable to propose that COX1 and 5-8-S rRNA are potentially relevant genes that affect the production of UFA and MUFA in the crossbreeding of Simmental and Mongolian cattle, but this needs to be further verified.

In this study, we have investigated Simmental cattle and their DEGs after crossbreeding with Mongolian and Holstein fattening cattle using transcriptome sequencing technology to reveal the effects of crossbreeding on gene expression and functional annotation of body metabolism. Examples include the glucose and muscle fatty acid content. We found that crossbreeding with Mongolian cattle may downregulation of PRSS2 increased GLU, thereby improving feed conversion efficiency. Therefore, we inferred that the downregulation of PRSS2 may have a synergistic effect with the increase in COX3, which together acted on GLU, which leads to a higher energy conversion capacity, and increased levels of the SM group GLU may lead to more NADH (47) entering the mitochondria and upregulating the expression of the COX3 gene, which may generate more energy through increased oxidative phosphorylation (48). Nevertheless, the expression of the COX3 gene was not validated in the present experiments, and these results need further real-time PCR (RT-PCR) or quantitative PCR (qPCR) validation.

## 5 Conclusion

In conclusion, based on our results, crossbreeding Simmental cattle with Mongolian cattle may downregulation of PRSS2 to increased GLU, thereby improving feed conversion efficiency, and may have a synergistic effect with the increase in COX3, which together acted on GLU, which leads to a higher energy conversion capacity, improving energy metabolism and providing more energy to the body in crossbred cattle. The benefits of hybrid dominance have been demonstrated and can be effective in meeting national breeding objectives. As a result, Crossbreeding with Mongolian cattle can increase the GLU metabolism of Simmental cattle, thus increasing the energy supply of Simmental cattle, with the aim of providing data and theoretical basis for improving the production performance of Simmental cattle. However, these results require further verification in order to provide a direction for future experiments.

## Data Availability

The datasets presented in this study can be found in online repositories. The names of the repository/repositories and accession number(s) can be found in the article/supplementary material.

## Footnotes

^1^http://asia.ensembl.org/Bos_taurus/Info/Index, ARS-UCD1.2
